# Effects of Developmental Activities and Interventions on Decision‐Making Skills in Soccer Players: A Scoping Review

**DOI:** 10.1111/sms.70250

**Published:** 2026-03-17

**Authors:** Guilherme Machado, Sixto González‐Víllora, Victor Reis Machado, Israel Teoldo

**Affiliations:** ^1^ Centre of Research and Studies in Soccer (NUPEF) Universidade Federal de Viçosa (UFV) Viçosa MG Brazil; ^2^ EDAF Research Group, Faculty of Education University of Castilla‐La Mancha Albacete Spain; ^3^ Scientific Department and Department of Athletes’ Integration and Development Paulista Football Federation (FPF) São Paulo Brazil

**Keywords:** coaching, cognition, football, profile history, sport pedagogy, training

## Abstract

Decision‐making is a key determinant of performance in soccer, yet evidence regarding how it develops across the sport pathway and how it can be effectively trained remains fragmented. This scoping review examined: (i) the effects of developmental activities and (ii) the effects of field‐based and laboratory‐based interventions on perceptual‐cognitive and perceptual‐motor decision‐making skills in youth and adult male and female soccer players. Following PRISMA and PRISMA‐ScR guidelines, six databases were systematically searched. Soccer players constituted the population, and outcomes included perceptual‐cognitive and perceptual‐motor decision‐making skills. Of 5,527 records identified, 36 studies met the inclusion criteria, with 50% published between 2020 and 2024. Seven studies addressed developmental activities, whereas 29 examined interventions. Evidence from developmental studies indicates that sustained engagement in soccer‐specific activities, particularly deliberate play and deliberate practice during childhood and early adolescence, is associated with higher decision‐making performance. Intervention studies showed that field‐based approaches, such as game‐based pedagogies, small‐sided games (SSG), and non‐linear training designs, can improve selected aspects of decision‐making across school, academy, and university contexts, although effects varied according to task design, assessment instruments, and intervention duration. In contrast, findings from laboratory‐based interventions were limited and inconsistent, with unclear transfer to on‐field decision‐making. Overall, the findings suggest that decision‐making development in soccer is influenced by both long‐term engagement in soccer‐specific activities and appropriately designed field‐based interventions. Key limitations include the predominance of male samples, heterogeneous methodologies, limited effect‐size reporting, and scarce research on decision‐making speed and female players. Directions for future research are outlined.

## 1 Introduction

The development of soccer players' ability to make efficient and quick decisions has become an important aspect of performance in contemporary soccer, due to changes in the dynamics of competitive matches and the increasing speed of play under reduced time and space constraints [1]. Empirical evidence has consistently shown that perceptual‐cognitive and perceptual‐motor decision‐making skills are associated with higher levels of performance and future success in soccer players [2, 3, 4, 5]. Reflecting these changes, the chief of *Global Football Development* for FIFA, Arséne Wenger, has stated that the next frontier in soccer is to train the players' brains and their decision‐making skills, leading FIFA to explicitly include, in its strategic agenda, the development of improved structural conditions and coach education programs aimed at fostering such skills [6]. Taken together, this body of evidence underscores that decision‐making is not only a key performance‐related attribute in soccer but also a central target for structured training and intervention efforts.

Decision‐making in sport has been divided into perceptual‐cognitive and perceptual‐motor skills, both contributing to the development of sport expertise [7, 8]. Perceptual‐cognitive skills can be described as what one perceives and understands, while perceptual‐motor skills refer to what one perceives and responds to through movement [9, 10]. In this context, decision‐making can be defined as the ability to select and execute the appropriate action based on the current circumstances on the field of play, as well as the strategic and tactical demands of the game [11, 12]. From an applied perspective, this conceptualization implies that decision‐making skills are not fixed attributes, but rather trainable capacities that can be shaped by the nature of players' experiences and the design of practice environments.

Research addressing the development of decision‐making skills in soccer has generally followed two complementary lines of inquiry. First, several studies have examined the role of developmental activities, such as deliberate play (e.g., playing soccer with friends in the park), deliberate practice (e.g., training with the team), and competition exposure, accumulated across childhood, adolescence, and adulthood, and their associations with later decision‐making performance [13, 14, 15]. These studies provide important insights into how long‐term participation patterns may contribute to the development of perceptual‐cognitive and perceptual‐motor skills, but they do not constitute interventions in the narrow sense. Second, an increasing body of research has focused on the effectiveness of structured interventions, implemented in either field‐based contexts (e.g., game‐based approaches, small‐sided games) or laboratory‐based settings, to directly target decision‐making processes [16, 17, 18, 19]. However, translating findings from intervention studies into applied coaching practice remains challenging due to substantial heterogeneity in study designs, outcome measures, contexts, and levels of ecological validity.

In recent years, several high‐quality systematic reviews and meta‐analyses have synthesized evidence on intervention‐based approaches relevant to decision‐making and skill acquisition in soccer and related contexts [20] conducted a soccer‐specific systematic review examining the influence of practice design and coaching behavior on perceptual‐motor and perceptual‐cognitive skill acquisition, with a strong emphasis on theoretical frameworks underpinning interventions conducted in applied settings. Complementing this work, Clemente et al. [21] synthesized evidence on the effects of small‐sided game interventions on technical execution and tactical behaviors in youth team sports, while Ortiz et al. [22] reported robust meta‐analytic evidence supporting the effectiveness of Teaching Games for Understanding approaches on decision‐making and game performance outcomes. More recently, Manninen et al. [23] provided a multilevel meta‐analysis comparing game‐based and traditional instructional approaches across physical education and sport contexts, highlighting both the potential benefits of game‐based approaches and the moderating role of contextual and methodological factors.

Despite these important contributions, the existing review literature remains fragmented in several key aspects [20, 21, 22, 23]. Previous reviews have predominantly focused on specific pedagogical models or types of interventions, often within either educational or sport‐specific contexts, without jointly considering how structured interventions relate to players' broader engagement in developmental activities across the sport development pathway [20, 22]. Moreover, no previous review has systematically mapped and contrasted evidence derived from field‐based and laboratory‐based interventions targeting decision‐making skills within an exclusive soccer‐specific scope, while simultaneously situating these findings in relation to players' long‐term developmental experiences [20, 23]. As a result, coaches, practitioners, and researchers currently lack an integrated overview of how different forms of practice exposure and intervention designs may contribute to the development of decision‐making skills in soccer.

Rather than providing additional evidence on isolated pedagogical models or single intervention types, a broader and integrative perspective is required to understand how decision‐making skills in soccer are developed across different forms of practice exposure [13, 15, 20, 24]. By jointly considering players' engagement in developmental activities over time and the effects of structured interventions implemented in both field‐based and laboratory settings, it becomes possible to clarify the scope, limits, and practical relevance of the existing evidence [23, 25, 26].

Such an integrative synthesis has implications beyond academic debate, as it may inform the design of coach education programs, support evidence‐based decision‐making by federations and governing bodies (e.g., FIFA, UEFA, CONMEBOL, CBF, RFEF, FA), and assist practitioners in aligning training environments with the cognitive and perceptual demands of the game [6, 27]. At the same time, mapping this body of literature can help identify priority areas for future research and contribute to a more efficient allocation of scientific, educational, and structural resources across the soccer development pathway. In addition to synthesizing the available evidence, this review uses an evidence gap‐map (EGM) to visually organize the body of research and to identify areas where evidence is abundant or scarce, thereby helping to highlight priority topics and guide future research in decision‐making development in soccer [28].

Therefore, in this scoping review, we aimed to investigate: (i) the effects of developmental activities and (ii) the effects of field‐based and laboratory‐based interventions on perceptual‐cognitive and perceptual‐motor decision‐making skills of youth and adult male and female soccer players.

## 2 Materials and Methods

### 2.1 Search Strategy and Inclusion Criteria

A scoping review of the available literature on developmental activities and interventions on decision‐making skills in soccer was conducted according to the Preferred Reporting Items for Systematic Reviews and Meta‐Analyses (PRISMA) guidelines [29], PRISMA extension for Scoping Reviews (PRISMA‐ScR) [30]. The Population, Intervention, Comparison, Outcome, Study design (PICOS) framework was adapted and employed to develop the search strategy. First, the population (P) of interest was soccer players. The intervention (I) under analysis was: (1) developmental activities in sport related to soccer activities, considering both (2) field‐based and laboratory‐based. No comparison (C) was used because the review sought to investigate what kind of interventions have been carried out in this topic. The outcome (O) was perceptual‐cognitive and perceptual‐motor decision‐making skills. The study design (S) is any empirical (either qualitative or quantitative) research.

In order to ensure article quality, six databases were used for the search: (1) Web of Science (all databases); (2) SCOPUS; (3) PubMed; (4) SPORTDiscuss (EBSCOhost); (5) PsycINFO; and (6) Scielo. The search was carried out for relevant publication prior to 6th June 2022 and updated on 29th December 2024. On the first level, the title, abstract, and keywords were searched through the following search equation: [(soccer OR football) AND (decision‐making OR “decision making” OR “response time” OR “response accuracy” OR “tactical awareness” OR “game performance” OR “game reading” OR game‐reading OR awareness OR perceptual‐cognitive OR “perceptual cognitive”) AND (intervention OR train* OR program OR pract* OR coaching OR “developmental activities” OR “history profile” OR development OR “deliberate practice” OR “deliberate play” OR play OR “early engagement” OR “specialized sampling” OR sampling OR diversification OR specialization) NOT (referee OR injur* OR “american football” OR “australian football”)]. Only empirical (either qualitative or quantitative) research was considered for further analysis.

The inclusion criteria for the articles were as follows: (1) published in peer‐reviewed scientific journals; (2) the validity and reliability of the instruments used to assess decision‐making in the studies should have been previously established and published in scientific journals; (3) written in English, Portuguese, or Spanish language. Therefore, the exclusion criteria were applied if the article: (1) was an intervention focusing on deleterious effects on decision‐making (e.g., mental fatigue and dehydration); (2) the intervention was not described in detail; and (3) was classified as low methodological quality (≤ 50%). In case of disagreement, it was solved by discussion between the two review authors.

Two independent reviewers (GM and VM) collected data from reports; being a systematic scoping review, data refers to study characteristics and their outcomes, but was not included in the actual results, which were not extracted. Disagreements were solved by discussion between the two reviewers.

A backward search was carried out through the screening of references for those selected articles in databases. Those references that exhaustively matched the inclusion criteria were included in the review.

### 2.2 Extraction of Data and Quality of the Studies

The quality of the studies was assessed with a risk‐of‐bias quality form (16 items) adapted from [31] and previously used in systematic reviews in sport [32, 33]. The items in the form assessed articles based on: objective (item 1); relevance of background literature (item 2); study design (items 3); sample included (items 4 and 5); informed consent obtention (item 6); outcome measures (items 7 and 8); description of methods (item 9); result significance (item 10); analysis methods (item 11); practical importance (item 12); drop‐outs description (item 13); appropriateness of conclusion (item 14); practical implications (item 15); and study limitations (item 16). The assessment for each item was a binary scale (1—meet criteria; 0—does not meet the criteria). The quality of the articles was expressed individually as a final score corresponding to the sum of the scores that met the criteria (1) divided by the total number of scored items (*n* = 16). Articles were classified based on their final scores as: low methodological quality (≤ 50%); good methodological quality (between 51% and 75%); and excellent methodological quality (> 75%) as used in previous studies [32, 34, 35].

We used a data extraction sheet (adapted from Cochrane Consumers and Communication Review Group's data extraction template). Initially, one of the researchers assessed the studies included in this review, and a second researcher checked the input data. Any disagreement was resolved by consensus between both researchers.

A narrative synthesis was performed, complemented by data summaries (number, percentage) for the previously defined data items. This approach involved the grouping of studies according to the type of decision‐making skills assessed and the type of situations assessed (e.g., offensive with the ball or defensive). Common themes were identified, and inferences were made about the data.

## 3 Results

### 3.1 Search, Selection, and Inclusion of Publications

**FIGURE 1:**
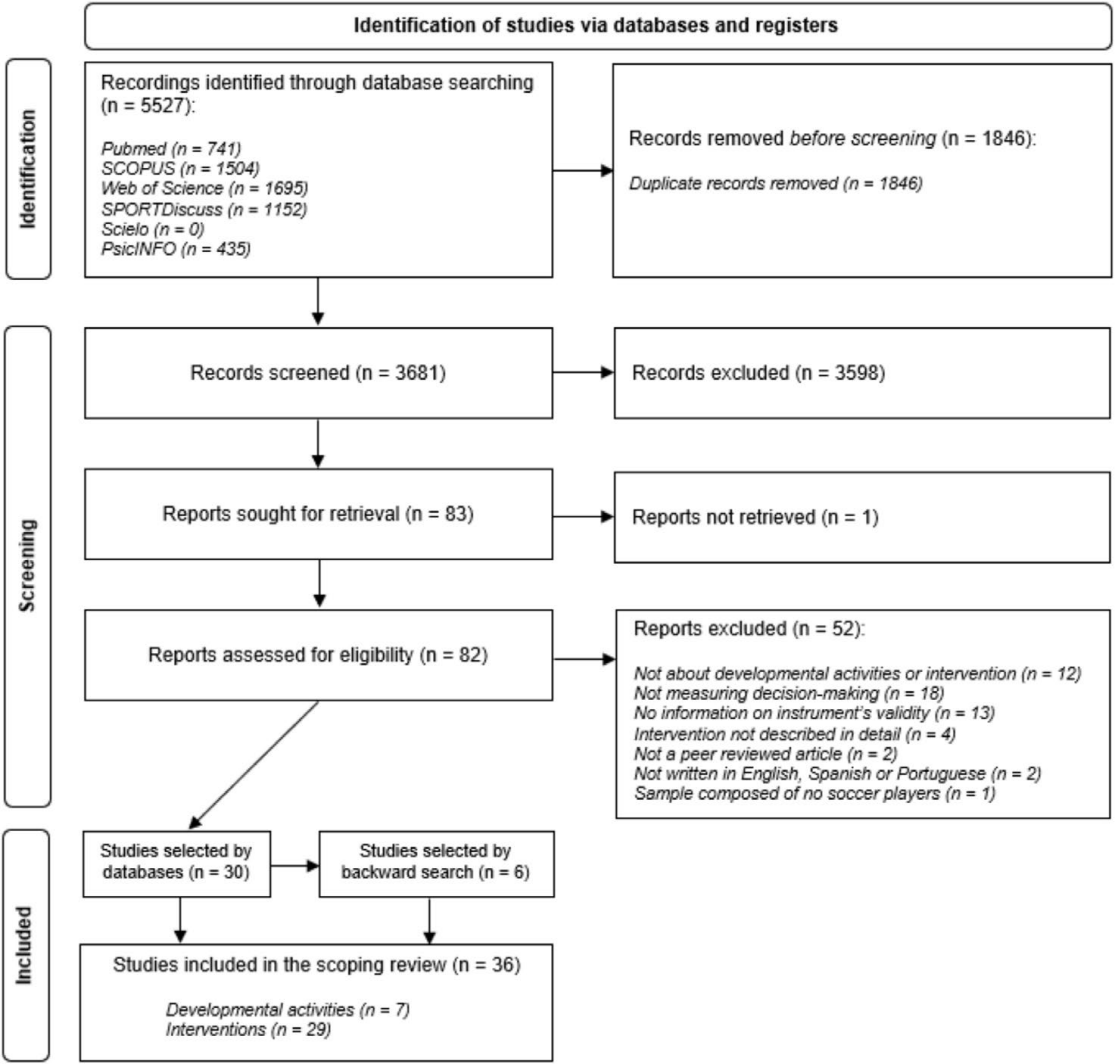
Flow chart of the methodology used for the article search based on the preferred reporting items for systematic review (PRISMA).

The study selection process is presented in Figure 1. Following database searching, duplicate removal, title and abstract screening, full‐text assessment, and backward reference searching, a total of 36 studies met the eligibility criteria and were included in the final synthesis, characterizing the current evidence base on decision‐making in soccer. All these procedures were conducted using a reference software manager (Mendeley Desktop, v. 1.19.8).

The temporal distribution of publications ranged from 2006 to 2024, revealing a clear increase in scientific output over time. Notably, 50% of the included studies were published between 2020 and 2024, indicating a growing and recent interest in decision‐making processes within soccer research.

**FIGURE 2:**
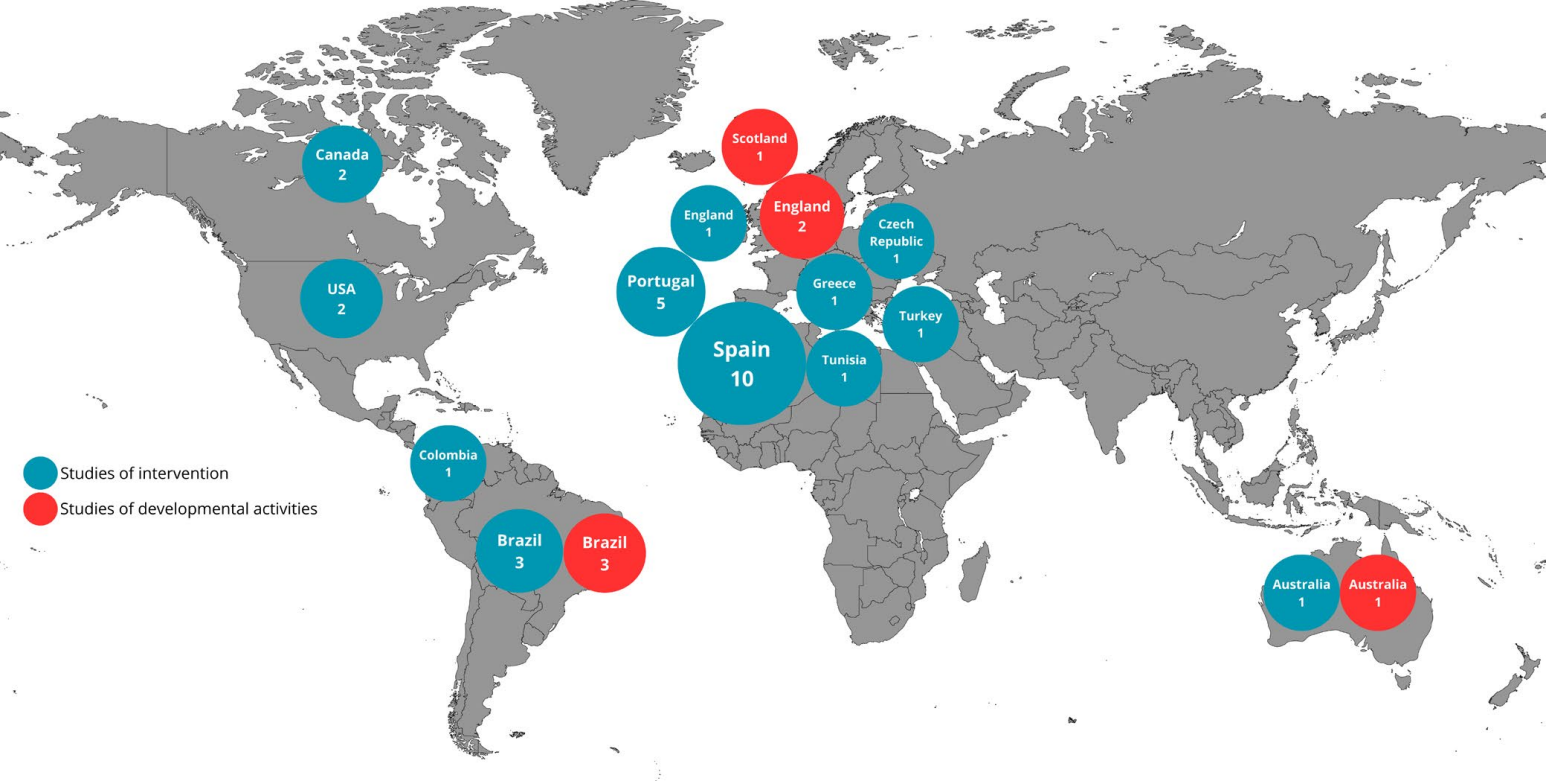
Distribution of the studies included per regions around the world.

**FIGURE 3:**
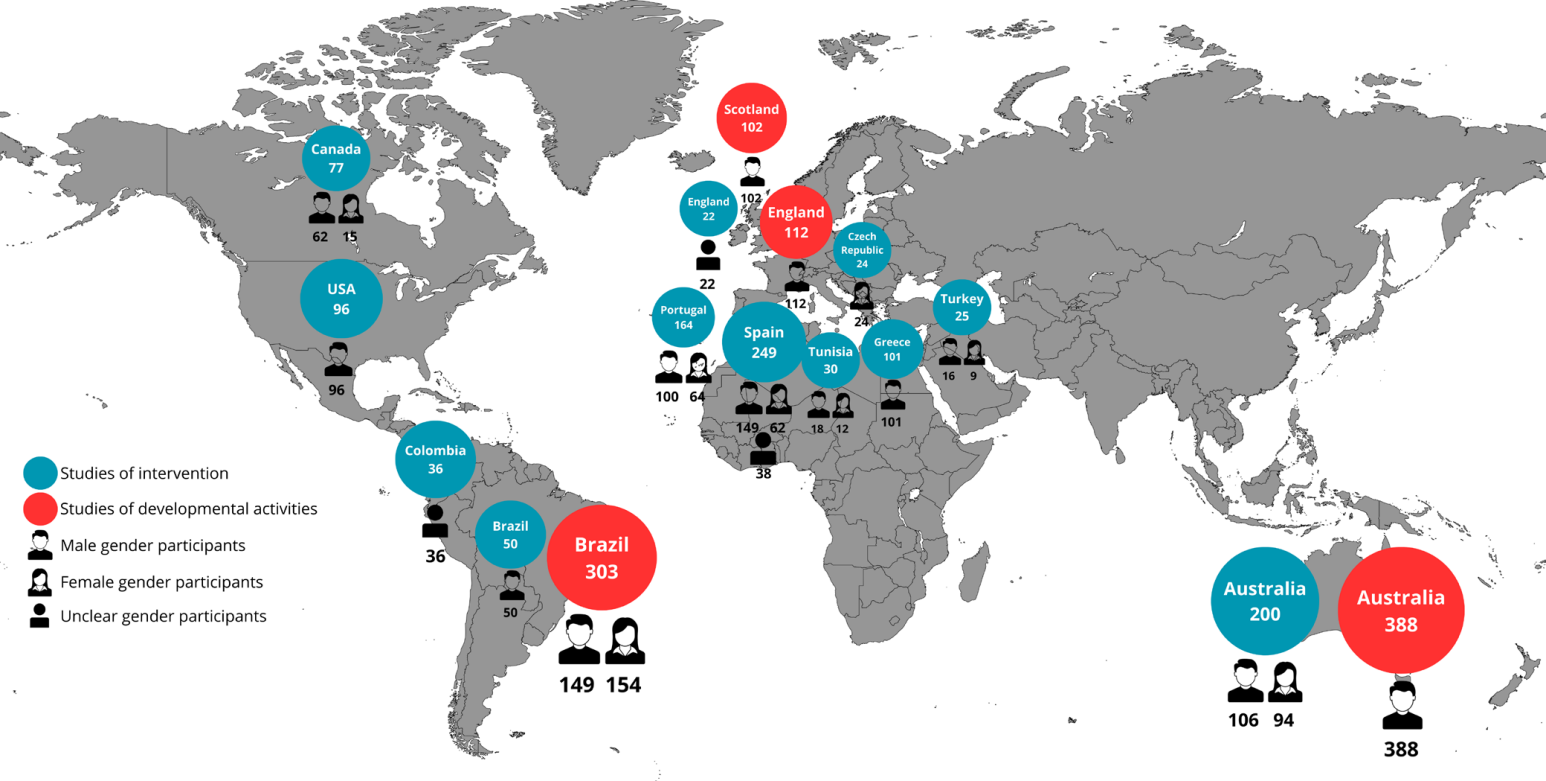
Distribution of the samples included per regions around the world.

The included studies were conducted across 13 countries, with a marked predominance of research originating from Europe, followed by South America and North America. Studies from Africa, Asia, and Oceania were scarce, highlighting an uneven global distribution of evidence (Figures 2 and 3). Although Oceania was represented by only a small number of studies, these investigations involved relatively large samples, resulting in one of the highest participant counts among the represented regions.

### 3.2 Quality of the Studies

Overall, the methodological quality of the included studies was high (Table 1). The vast majority of studies achieved an excellent methodological quality rating, while only a small proportion were classified as having good quality, indicating a generally robust evidence base.

**TABLE 1:** Studies with the main topic of developmental activities.

Article information		Participants information				Decision‐making assessment information				Developmental activities information		
Author	Quality	Participants (M ± SD)	Country of the sample	Context	Gender	Decision‐making assessed	Methodology & Instrument	Situations assessed	Type of variable	Developmental activities/milestones assessed	Periods Analyzed	Main results
Machado et al. [25, 36]	94%	*N* = 77 (23.4 ± 4.2); high decision‐making skills: *n* = 25; low decision‐making skills: *n* = 25	Brazil	Adult—soccer club level	Female	PCS	Video—TacticUP	OwB; OwfB; Def	Quality	Practice in soccer; play in soccer; percentage of time in play; practice in futsal; total practice (soccer + futsal); number of other sports.	Childhood (6–12 years); early adolescence (13–15 years); adolescence (13–18 years)	The high decision‐making skill group spent more time participating in developmental activities in every period that was assessed (childhood, early, and late adolescence) compared with their low decision‐making skill group. In summary, the high decision‐making skill group accumulated more percentage time of participating in soccer activities in practice in every period that was assessed; and gathered more hours of practice in futsal and total practice in early adolescence (13–15 years), compared to the low decision‐making skills group
Larkin et al. [37]	94%	*N* = 388 (13.8 ± 0.8)	Australia	Adolescent—soccer club level	Male	PCS	Video—PCT	OwB	Quality	Coach‐led practice; competition; self‐led practice; play with pees; indirect involvement.	Whole career (8 ~ 13.8)	Youth soccer players who display consistent interest tend to be more skilled and accumulate considerably more highly structured and effortful practice than their less gritty peers. The accumulated structured practice then determines the level of perceptual‐cognitive ability because the players who spent more time on soccer related activities demonstrated higher levels of perceptual‐cognitive skill
Roca and Ford [15]	94%	*N* = 48 (20.2 ± 2.1); high‐creative: *n* = 12; low‐creative: *n* = 12	England	Adult—soccer club level	Male	PCS	Video—SSCT	OwB	Quality	Practice; play; competition/start age in: soccer; supervised training; soccer league; elite training program; semi‐professional level; professional level.	Childhood (6–12 years); early adolescence (13–15 years); adolescence (13–18 years)	High skill group spent more hours per year in deliberate play during childhood and early adolescence (i.e., 6–15 years of age) compared with their low‐skill counterparts. No differences were reported for hours spent in deliberate practice and competition nor in milestones
Machado et al. [38]	88%	*N* = 77 (23.4 ± 4.2); high decision‐making skills: *n* = 25; low decision‐making skills: *n* = 25	Brazil	Adult—soccer club level	Female	PCS	Video—TacticUP	OwB; OwfB; Def	Quality; Speed	Practice in soccer; play in soccer; percentage of time in play; practice in futsal.	Childhood (6–12 years); early adolescence (13–15 years); adolescence (13–18 years)	Engagement in deliberate practice in soccer and futsal, especially during childhood and early adolescence, is related to a better quality of offensive decision‐making skills. Furthermore, deliberate practice in futsal is associated only with offensive decision‐making skills with the ball and near the ball. In turn, engaging in deliberate play in soccer, mainly in childhood and early adolescence, is related to quicker offensive and defensive decision‐making skills.
Machado et al. [14]	88%	*N* = 149 (14.9 ± 1.6); high‐skill: *n* = 50; low‐skill: *n* = 50	Brazil	Adolescent—soccer club level	Male	PCS	Video—TacticUP	OwB; OwfB; Def	Quality	Macrostructure: team practice; individual practice; play; competition. Microstructure of team practice: individual; pair; drills; group tactics; collective tactics; low decision‐making opportunities; high decision‐making opportunities; players'perceived opportunities to make decisions.	Whole career (6 ~ 14.9)	The main activities that differentiated high−/low‐skill groups in both offensive and defesive decision‐making skills in the macrostructure activities were: (i) team practice; and in the microstructure: (ii) collective tactics; and (iii) “high decision‐making opportunities”. The high‐skill group accumulated more hours in the aforementioned activities
Hendry et al. [13]	88%	*N* = 102 (14.8 ± 0.6); academy‐only: *n* = 76; youth‐professional only: *n* = 17; adult‐professional: *n* = 9	Scotland	Adolescent—soccer club level	Male	PMS	Subjective Scale—Skill rating	OwB, OwtB, Def	Quality	Practice; play; play %.	Childhood (5–12 years); whole career (5 to 16–18 years)	For players that transitioned to youth‐professional, creative decision‐making skill ratings at T2 (16–18 years) positive correlated to childhood practice and whole career practice. Moreover, tactical decision‐making skill ratings negatively correlated to play % during childhood and positively correlated to practice during the whole career
Roca et al. [24]	81%	*N* = 64 Skilled: *N* = 48 (20.7 ± 2.4) Recreational: *N* = 16 (22.1 ± 2.8)	United Kingdom	Adult—soccer club & recreational level	Male	PCS	Video—PCT	OwB	Quality	Practice; play; competition/start age in: soccer; supervised training; soccer league; elite training program; semi‐professional level.	Childhood (6–12 years); adolescence (13–18 years)	Play in soccer during childhood was the strongest predictor of perceptual‐cognitive expertise. Soccer practice during adolescence was also a predictor, albeit its impact was relatively modest. Competition and milestones showed no differences

Abbreviations: Def, defensive; NR, not reported; OwB, offensive with the ball; OwtB, offensive without the ball; PCS, perceptual‐cognitive skills; PCT, perceptual‐cognitive test; Play %, percentual time in play considering the total time spent in play and practice activities; PMS, perceptual‐motor skills; SSCT, soccer‐specific criativity test.

Despite this favorable methodological profile, two recurring limitations were consistently identified across studies: insufficient reporting of participant dropouts and a lack of justification for sample size determination. These issues may limit transparency and reproducibility, despite the overall strong methodological standards observed.

### 3.3 Study Characteristics

Key characteristics of the included studies are summarized in Tables 1 and 2. Overall, the evidence base was dominated by intervention studies (*n* = 29), with a smaller subset focusing on developmental activities (*n* = 7). Across all studies, nearly 2000 participants were involved, with samples predominantly composed of male players, although female players were also represented in a limited number of investigations.

**TABLE 2:** Studies with the main topic intervention.

Article information		Participant information			Decision‐making assessment information				Intervention information						
Authors	Quality	Participants (M ± SD)	Context	Gender	Decision‐making assessed	Methodology & Instrument	Situations assessed	Type of variable	Total sessions	Duration (w)	Sessions per week	Time per session (m)	Groups	Intervention/Control Effectiveness	Main results
**Studies with school players**															
Farias et al. [39]	88%	*N* = 26 (12.3); boys = 16; girls = 10	Children—school	Male & female	PMS	SSG—GPAI	OwB; OwtB; Def	Quality	16	NR	2	45	Intervention: Sport Education (SE) + Invasion Games Competence model (IGCM)	↑	The intervention improved decisions on situations of creating scoring opportunities and overall game performance
Farias et al. [40]	81%	*N* = 24 (10.3 years); boys = 16; girls = 8	Children—school	Male & female	PCS; PMS	SSG & Questionnaire—GPOI & GUT	OwB, OwtB, Def	Quality	17	NR	2	45–90	Intervention: Sport Education (SE) + Invasion Games Competence model (IGCM)	↑	PCS improved on defensive decisions for girls and offensive decisions for girls and boys. PMS improved on defensive and offensive decisions for girls and boys
Mesquita et al. [41]	88%	*N* = 26 (10–12 years); boys = 17; girls = 9	Children—school	Male & female	PMS	SSG—GPAI	OwB, OwtB, Def	Quality	22	NR	NR	NR	Intervention: Sport Education (SE) + Invasion Games Competence model (IGCM)	↑	Defensive and offensive decisions improved for girls, but not for boys. Groups of low, medium, and high skill levels developed defensive decision skills, but not offensive
Farias et al. [42]	88%	*N* = 26 (12.3 ± 1.3); boys = 16; girls = 10	Children—school	Male & female	PMS	SSG—GPAI	OwB; OwtB; Def	Quality	18	9	2	45	Intervention: Sport Education (SE)	↑	The SE intervention improved game performance in soccer
Dervent et al. [43]	81%	*N* = 27 (12.44 ± 0.64); boys = 16; girls = 9; Intervention 1: *N* = 14; Intervention 2: *N* = 13	Children—school	Male & female	PMS	SSG—Specific coding	OwB; OwtB; Def	Quality	10	5	2	40	Intervention 1: Situated Game Teaching through Set Plays (SGTSP); Intervention 2: Technique‐focused group (TFG)	↑/↑	The SGTSP group improved decision‐making more than TFG
Garcia‐Ceberino et al. [44]	81%	*N* = 35 (10.6 ± 0.5); boys = 19; girls = 16; Intervention: *N* = 17; Control: *N* = 18	Children—school	Male & female	PMS	SSG—IMLPFoot	OwB; OwtB; Def	Quality	9	NR	NR	40	Intervention: Tactical games approach soccer (TGAS); Control: Direct instruction soccer (DIS)	→/→	Decision‐making skills showed no improvements in both intervention programs
Gouveia et al. [45]	94%	*N* = 62 (13–16 years); boys = 35; girls = 27; Intervention: *N* = 41; Control: *N* = 21	Adolescent—school	Male & female	PMS	SSG—GPAI	Owb; OwtB	Quality	24	8	3	45	Intervention 1: Tactical games approach (TGA) of soccer, handball and basketball; Control: Technique approach (TA) of soccer, handball and basketball	↑/↑	Both TGA and TA groups improved their on‐the‐ball decision‐making. No differences were found for effectiveness of these two approaches
Chatzopoulos et al. [46]	81%	*N* = 101 (12–13 years); Intervention 1: *N* = 49; Intervention 2: *N* = 52	Children & adolescent—school	Male	PMS	SSG—Modified GPAI	Owb	Quality	15	5	3	30	Intervention 1: Games approach (GA); Intervention 2: Technique approach (TA)	↑/→	Decision‐making skills in situations with the ball improved in the GA group, but not in the TA group
Khalifa et al. [47]	69%	*N* = 30 (15.4 ± 0.6); boys = 18; girls = 12	Adolescent—school	Male & female	PMS	SSG—GPAI	OwB; OwtB; Def	Quality	12	6	2	60	Intervention: Verbal interaction during training (play–discuss–play); Control: non‐verbal interaction during training (play–passive recovery–play)	↑/↑	Both groups improved their decisions from pre‐ to post‐test, but the verbal interaction group had greater improvement
Whipp et al. [48]	88%	*N* = 200 (12.5 ± 0.6); boys = 106; girls = 94; Intervention: *N* = 94; Control: *N* = 106	Children—school	Male & female	PMS	SSG—GPAI	OwB; OwtB; Def	Quality	10	5	2	30	Intervention: Trained peer‐teachers (T‐PT); Control: Untrained peer‐teachers (U‐PT)	↑/→	Game performance improved for the T‐PT group, but not for the U‐PT group
Viciana et al. [49]	94%	*N* = 85 (10.6 ± 0.6); boys = 39; girls = 46; Intervention: *N* = 43; Control: *N* = 42	Children—school	Male & female	PMS	SSG—GPAI	OwB, OwtB, Def	Quality	10	8	2	60	Intervention: Alternated teaching units of soccer and basketball (ATU); Control: Traditional teaching units of soccer and basketball (TTU)	→/→	The ATU group had a higher improvement in the decision‐making index in overall invasion sports compared to the TTU group, but not in soccer
**Studies with soccer academy players**															
Sierra‐Ríos et al. [50]	94%	*N* = 30; Intervention: *N* = 15 (10.0 ± 0.1); Control: *N* = 15 (10.6 ± 0.6)	Children—soccer club	Male	PMS	SSG—GPET	OwB; OwtB	Quality	12	6	2	80	Intervention: Teaching Games for Understanding (TGfU); Control: Direct Instruction (DI)	↑/→	Decision‐making skills improved for the TGfU group in offensive actions off the ball, but not for actions with the ball, while no improvement for the DI group was found
Práxedes et al. [51]	88%	*N* = 9 (10.6 ± 0.5)	Children—Soccer club	Male	PMS	SSG—GPET	OwB	Quality	22	12	2	60	Intervention: Teaching Games for Understanding (TGfU)	↑	Total decision‐making skills and decisions in passing actions improved, while no improvement was found for decisions in dribbling actions
Práxedes et al. [27]	88%	*N* = 18 (10.7 ± 0.6); Intervention: *N* = 9; Control: *N* = 9	Children—soccer club	Male	PMS	Competition Match—GPET	OwB	Quality	21	12	2	60	Intervention: Teaching Games for Understanding (TGfU); Control: Technique approach (TA)	↑/→	The TGfU group improved decision‐making skills related to passing and dribbling situations, compared to the TA group
Harvey et al. [52]	81%	*N* = 34 (14–18 years); High‐skill (varsity) = 18 (14–18 years); Low‐skill (first year) = 16 (14–15 years)	Adolescent—soccer club & recreational	Male	PMS	SSG—GPAI	OwB; Def	Quality	8	8	1	45–60	Intervention: Teaching Games for Understanding (TGfU)	→	The TGfU intervention group did not improved the number of correct decisions, although it decreased the number of inappropriate decisions in the post‐test for the high‐skill group, but not for the low‐skill group
Roberts et al. [53]	88%	*N* = 22; Intervention: *N* = 11 (16.4 ± 0.4); Control: *N* = 11 (16.1 ± 0.2)	Adolescent—soccer club	NR	PMS	Skill test—LSST	OwB	Quality; Speed	8	4	2	60	Intervention: “pitch‐based” non‐linear pedagogy training (NLP); Control: “pitch‐based” linear pedagogy training (LP)	↑/→	Improvement in decision‐making in a shooting task was found for the NLP group, but not for the LP group
Práxedes et al. [26]	81%	*N* = 19 (10.6 ± 0.5); Intervention: *N* = 10; Control: *N* = 9	Children—soccer club	NR	PMS	Competition Match—GPET	OwB	Quality	14	7	2	60	Intervention: Non‐linear pedagogy (NLP); Control: Direct instruction model (DIM)	↑/→	Decision‐making skills related to passing situations improved with the NLP group
Práxedes et al. [54]	81%	*N* = 19; Intervention: *N* = 10 (10.6 ± 0.5); Control: *N* = 9 (11.8 ± 0.7)	Children—soccer club	Male	PMS	Competition Match—GPET	OwB	Quality	14	7	2	60	Intervention: Non‐linear pedagogy (NLP); Control: Direct instruction model (DIM)	↑/→	The NLP group improved decision‐making skills related to passing situations, but not in dribling situations
Barcellos et al. [55]	88%	*N* = 25 (12.0 ± 0.2)	Children—soccer club	Male	PCS	Video—TacticUP	OwB; OwtB; Def	Quality	25	7	4	28–50	Intervention: SSG based on core tactical principles and players´ interaction	↑	Decision‐making skills improved related to offensive tactical principles far from the ball
Machado et al. [14]	88%	*N* = 25 (12.0 ± 0.2)	Children—soccer club	Male	PCS; PMS	SSG & Video—FUT‐SAT & TacticUP	OwB; OwtB; Def	Quality; Speed	25	7	4	28–50	Intervention: SSG based on core tactical principles and players´ interaction	↑	Perceptual‐cognitive decision‐making time for both offensive and defensive actions performed inside the centre of play and defensive actions performed outside the centre of play were improved. Moreover, it was also found improvement in perceptual‐motor skills for defensive actions performed inside the centre of play
Romeas et al. [56]	94%	*N* = 62 (15.36 ± 0.24); U13 = 22; U14 = 16; U16 = 16; U18 = 14; Intervention: *N* = 30; Control: *N* = 32	Adolescent—soccer club	Male	PMS	SSG—GPAI	OwB; OwtB	Quality	26 ~ 56	10	1 ~ 3	NR	Intervention: 3D MOT + BMP + 3D MOT BMP; Passive Control: none	→	There was no transfer of the improvement in the 3DMOT training program to decision‐making in on‐field performance.
Feria‐Madueño et al. [57]	94%	*N* = 20 (8.5 ± 1.); Intervention: *N* = 10; Control *N* = 10	Children—soccer club	Male	PMS	SSG—GPET	OwB; OwtB; Def	Quality	12	6	2	15	Intervention: Attention training using a video‐game; Control: None	↑/→	Attention training using a video‐game group improved decision‐making skills related to shooting and marking with the ball more than their counterparts in the control group.
Villaseca‐Vicuña et al. [58]	88%	*N* = 10 U16 players (14.4 ± 0.5)	Adolescent—soccer club	Male	PMS	Skill test—LSST	OwB	Speed	6	6	1	5	Intervention 1: Integrated warm‐up protocol (IWP); Intervention 2: Analytical warm‐up protocol (AWP)	↑/→	Players displayed greater technical‐decisional performance in LSPT after executing the IWP
Valencia‐Sánchez and Arias [59]	88%	*N* = 36 (10.0 ± 0.6)	Children—soccer school	NR	PMS	SSG—FUT‐SAT	OwB; OwtB; Def	Quality	12	12	1	80	Intervention: Didactic model game action competences (DMGAC); Control: Didactical model of direct instruction (DMDI)	↑/→	Participants in DMGAC attained a higher tactical performance index than those who participated in DMDI in the post‐test.
Práxedes et al. [60]	88%	*N* = 19; Intervention: *N* = 10 (10.6 ± 0.5); Control: *N* = 9 (10.7 ± 0.5)	Children—soccer club	NR	PMS	Competition Match—GPET	OwB	Quality	14	7	2	60	Intervention 1: SSG with numerical superiority (SSGNS); Intervention 2: SSG with numerical equality (SSGNE)	↑/→	Decision‐making skills related to passing situations improved in the SSGNS intervention with the average skill‐level group, but not for the SSGNE intervention or the low skill‐level group
**Studies with university players**															
Harenberg et al. [61]	100%	*N* = 31; Intervention: *N* = 16 (19.3 ± 1.1); Control: *N* = 15 (18.9 ± 0.6)	Adult—university	Male & female	PCS	Video—Soccer‐specific motor response	OwB	Quality	10	4	NR	25–30	Intervention: 3D MOT; Control: Watched soccer scenes	→/→	Non‐significant changes in decision‐making were found. The results did not confirm an association of 3D MOT and decision‐making in soccer
Romeas et al. [62]	88%	*N* = 23; Intervention: *N* = 9 (21.3 ± 0.8); Active control: *N* = 7 (21.4 ± 1.0); Passive control: *N* = 7 (22.5 ± 0.7)	Adult—university	Male	PCS; PMS	SSG & Questionnaire—Specific coding & SPSA	OwB	Quality	10	5	2	NR	Intervention: 3D MOT; Active Control: Watched soccer scenes; Passive Control: none	↑/→	Perceptual‐motor decision‐making accuracy in passing improved for the 3D‐MOT group compared to control groups, but not in dribbling and shooting. Subjective perceptual‐cognitive decision‐making accuracy also improved in the 3D‐MOT group compared to control groups
Psotta and Martin [63]	75%	*N* = 24; Intervention: *N* = 12 (20.7 ± 0.8); Control: *N* = 12 (21.0 ± 0.7)	Adult—University	Female	PMS	Competition Match—SPOS	OwB	Quality	10	5	2	90	Intervention: Combined tactical model (CTA); Control: Combined technical model (CTE)	↑/↑	The decision making index was enhanced after intervention in both CTA and CTE groups. Decision‐making in passing situations improved in the CTA group, but not in dribbling and shooting
**Studies with adult players**															
Fortes et al. [64]	100%	*N* = 23 (22.6 ± 2.3); Intervention: *N* = 11; Sham: *N* = 12	Adult—professional	Male	PCS; PMS	SSG & Video—Specific coding & SSDMT	OwB	Quality; Speed	40	8	5	15	Intervention: anodal transcranial direct current stimulation (a‐tDCS); Sham: (a‐tDCS) turned off in 30 s	↑/→	The a‐tDCS group significantly reduced the response time in the decision‐making test. No changes in decision‐making accuracy were found

Abbreviations: ↑, significant increase; →, no significant difference; Def, defensive; FUT‐SAT, System of Tactical Assessment in Soccer; GPAI, Game Performance Assessment Instrument; GPET, Game Performance Evaluation Tool; GPOI, Game Performance Observation Tool; GUT, Game Understanding Test; IMLPFoot, Instrument for the Measurement of Learning and Performance in Football; LSST, Loughborough shooting skill test; NR, not reported; OwB, offensive with the ball; OwtB, offensive without the ball; PCS, perceptual‐cognitive skills; PMS, perceptual‐motor skills; SPOS, Soccer Performance Observation System; SPSA, Sport Performance Scale application; SSDMT, Soccer Specific Decision‐making Test.

Most investigations were conducted in soccer clubs and school settings, highlighting the applied and developmental orientation of the field. With regard to age categories, research focused mainly on children and adolescents, whereas adult populations were comparatively underrepresented. This distribution suggests a strong emphasis on early and mid‐developmental stages in decision‐making research.

### 3.4 Type of Decision‐Making Assessed

Most studies assessed decision‐making through perceptual‐motor approaches, whereas fewer focused on perceptual‐cognitive processes or combined both dimensions. Similarly, the assessment of decision‐making quality was far more prevalent than the assessment of decision‐making speed.

**FIGURE 4:**
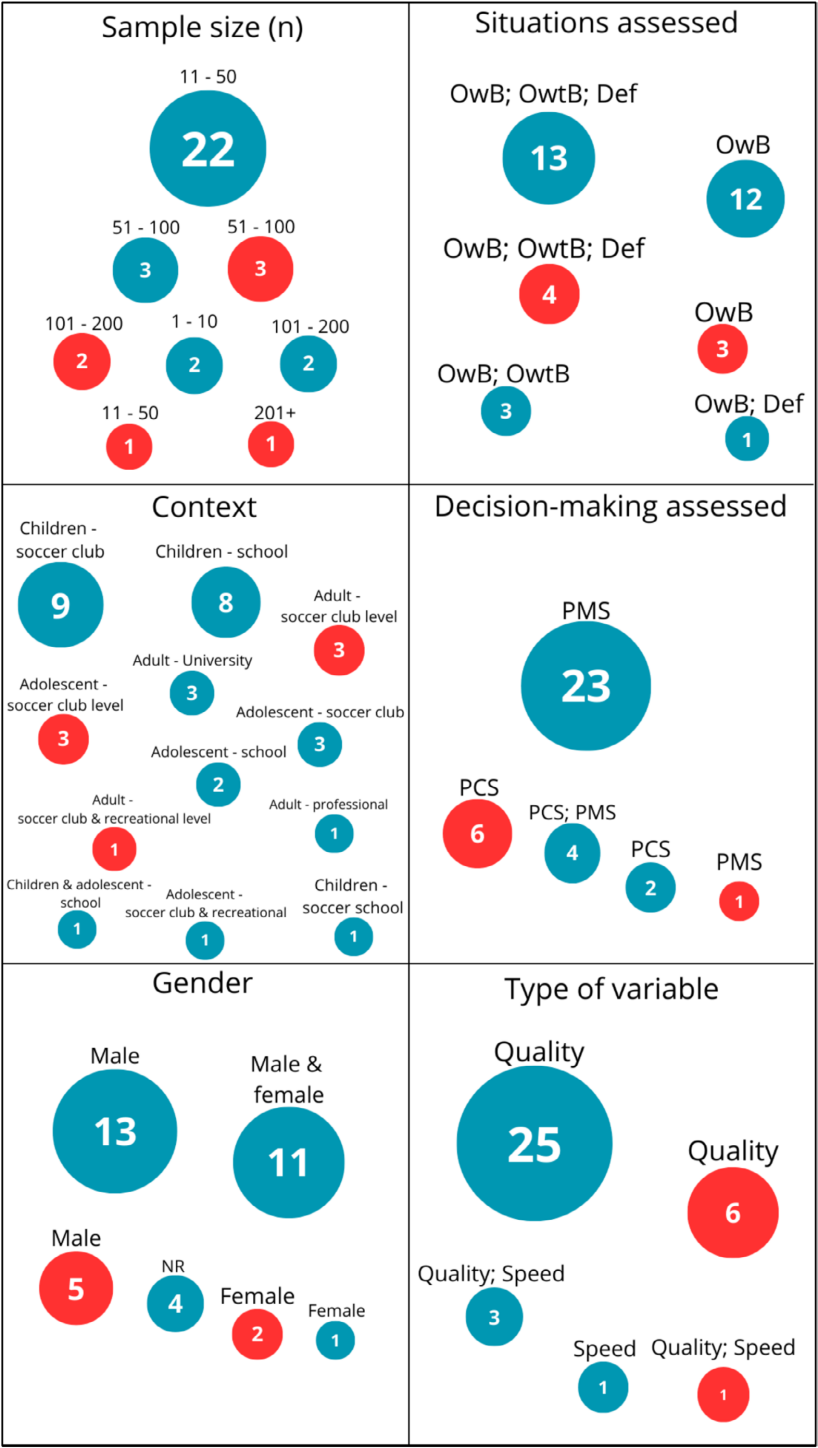
Evidence gap‐map of studies of expertise and interventions with decision‐making in soccer players. Def, defensive; OwB, offensive with ball; OwtB, offensive without the ball; PCS, perceptual‐cognitive skills; PMS, perceptual‐motor skills.

Figure 4 presents an evidence gap‐map summarizing the characteristics of the studies included in this review. On the left side of the figure, the map displays the sample‐related characteristics of the studies, including sample size, study context, and participant gender. On the right side, the figure summarizes characteristics related to how decision‐making was assessed, including the decision‐making situation evaluated (offensive with the ball, offensive without the ball, and defensive), the decision‐making assessed (perceptual‐motor, perceptual‐cognitive, or both), and the type of outcome variable measured (quality, speed, or both). This visualization provides an integrated overview of both the populations investigated and the methodological approaches used to assess decision‐making in soccer research. Overall, the map indicates a predominance of studies focusing on perceptual‐motor decision‐making and decision‐making quality, whereas fewer investigations have examined perceptual‐cognitive processes, decision‐making speed, or a broader range of contexts and participant characteristics. This evidence gap‐map also highlights areas that remain underrepresented in the literature.

### 3.5 Developmental Activities

Studies examining developmental activities primarily focused on players' engagement in deliberate practice and deliberate play, alongside competitive participation and sport‐specific milestones. These variables constituted the core developmental factors examined across studies, whereas other forms of developmental exposure, such as self‐regulated practice, participation in other sports, or indirect involvement (e.g., video games), were rarely investigated. This pattern reflects a relatively narrow conceptualization of developmental pathways within the existing literature (Table 1).

### 3.6 Interventions

A wide range of pedagogical, tactical, and cognitive interventions was identified among the intervention studies (Table 2). Most interventions were grounded in game‐based pedagogical models and SSG, while a smaller number explored perceptual‐cognitive training programs or technology‐ and neurophysiology‐based approaches.

Control conditions were similarly heterogeneous, most commonly involving direct instruction or traditional technical approaches. Overall, this diversity of intervention designs illustrates the multidimensional nature of decision‐making training in soccer, while also underscoring the challenges associated with directly comparing outcomes across studies.

## 4 Discussion

This scoping review aimed to investigate: (i) the effect of developmental activities and (ii) the effect of field‐based and laboratory‐based interventions on perceptual‐cognitive and perceptual‐motor decision‐making skills of youth and adult male and female soccer players. Overall, studies about developmental activities focused mainly on the development of perceptual‐cognitive skills (86%) with a majority of male samples (61%), while most studies about intervention assessed only perceptual‐motor skills (83%). Regarding quality, almost all studies included in this scoping review possessed excellent quality (92%).

### 4.1 Developmental Activities

The nature of the evidence derived from studies examining developmental activities is primarily based on retrospective or correlational designs that describe players' accumulated engagement in different soccer‐related activities during childhood, adolescence, and adulthood. As such, these studies do not involve the systematic manipulation of training variables or controlled exposure to specific practice conditions. Consequently, their findings should be interpreted as indicative of developmental associations and plausible learning mechanisms across time, rather than as effects derived from experimentally controlled intervention designs, including field‐ or laboratory‐based intervention studies.

The studies carried out with developmental activities showed the contribution of different types of activities for developing decision‐making skills across different phases of sports formation (e.g., childhood and adolescence). Deliberate play undertaken in childhood (i.e., 6–12 years) was positively related to the quality of defensive perceptual‐cognitive decision‐making skills [24], speed of defensive decision‐making skills [65], offensive creative decision‐making skills [24], and speed of offensive decision‐making skills [65], measured through objective video‐based tests. Moreover, deliberate play during early adolescence (i.e., 13–15 years) was positively associated with offensive creative decision‐making skills [15] and speed of offensive and defensive decision‐making skills [65] assessed in adulthood. On the other hand, other studies [13] found no association between deliberate play reported across childhood and adolescence with perceptual‐motor decision‐making skills related to tactical and creative actions, although it was assessed through a subjective coaching rate scale.

One explanation for the contribution of deliberate play to better decision‐making skills is its nature, which usually couples perceptual‐cognitive and perceptual‐motor skills in unstructured activities [66]. Those activities usually can be characterized by (1) mimicking game‐like sub‐phases situations (e.g., 2 × 2, 3 × 3); (2) providing modified tasks and environmental constraints (e.g., smaller and irregular playing fields); and (3) having variability of practice in uncertain contexts (e.g., requiring many soccer skills to be executed in a random format). Such practice characteristics induce athletes to provide quick solutions for game problems and provide a stimulus for players to develop skills related to creative and intelligent decision‐making [15].

Considering the deliberate practice, studies showed a positive association with perceptual‐cognitive decision‐making skills in defensive [14, 24] and offensive [14, 65] situations, during childhood (i.e., 6–12 years) [65], early adolescence (i.e., 13–15 years) [65], adolescence (i.e., 13–18 years) [24, 65], and childhood plus early adolescence altogether (i.e., 6 to ~15 years) [14, 37]. Moreover, a positive correlation was found between deliberate practice with perceptual‐motor decision‐making skills related to creative actions during childhood [13] and related to tactical and creative actions during childhood plus adolescence altogether (i.e., 5 to ~17 years) [13]. In this regard, involvement with structured activities with its primary aim of developing performance looks to be an important aspect for athletes to develop their decision‐making quality through their sport formation [67]. This context usually provides athletes with exercises focusing on performance improvement, with valuable feedback from the coaching staff [68]. Moreover, a recent study found that soccer practice has been changing throughout the last 15 years to provide players nowadays with higher opportunities to make decisions [68] compared to practice prior to 2010 [69]. Such a practice characteristic has been associated with the acquisition of better offensive and defensive decision‐making quality in soccer [14].

Recent research [38, 65] further refines our understanding of this interplay regarding the balance between play and practice. Several retrospective studies have sought to quantify the proportion of accumulated hours spent in play and practice across development by deriving an index of relative engagement between these activities (i.e., “% time in play”) [13, 25, 36, 38, 65, 70]. Findings indicate that a more balanced distribution of engagement during childhood (e.g., approximately 50% play/50% practice) is positively associated with higher decision‐making performance in adulthood, compared with more polarized patterns favoring either practice (e.g., ~70% practice/30% play) or play [38, 65]. As players transition into early adolescence (13–15 years), this balance appears to shift, with elite players progressively increasing their engagement in structured practice, suggesting that the relative contribution of play and practice evolves as functional demands and learning needs change over time [25, 36, 38, 70]. From a developmental perspective, this pattern reflects the idea that decision‐making skill acquisition emerges from the ongoing interaction between the learner, the task, and the performance environment, rather than from a fixed sequence of activities. In line with this perspective, O'Sullivan et al. [71] emphasize that effective player development depends on the dynamic alignment of developmental stage, practice design, and contextual influences, reinforcing the notion that the play–practice balance should adapt across the developmental pathway rather than conform to a static or linear model.

In terms of the contribution of other types of activities, albeit deliberate play and practice, the study of Machado et al. [14] that analyzed perceptual‐cognitive decision‐making skills in the period of childhood plus early adolescence altogether (i.e., 6 to ~15 years) showed a positive association between: (i) competition with offensive skills; (ii) individual practice with defensive skills; and (iii) the microstructure of practice such as activities of group tactics (i.e., 2 × 2 up to 4 × 4) and collective tactics (i.e., 5 × 5 up to 11 × 11) with offensive and defensive skills. Although positive associations have been reported for individual practice, the broader literature indicates that this type of activity is more closely related to motor and technical development than to perceptual‐cognitive skill acquisition per se [12, 67], and its relationship with decision‐making may therefore reflect the broader structure of deliberate practice in soccer, which integrates individual and collective training components [25, 36, 38, 69, 70].

Furthermore, practice in futsal during childhood and early adolescence has been linked to enhanced perceptual‐cognitive decision‐making skills, especially in decision‐making skills related to offensive situations with the ball and near the ball [38, 65] assessed in adulthood. On the other hand, other studies showed no association between perceptual‐cognitive decision‐making skills and milestones (e.g., start age at playing and practicing soccer) nor the number and hours accumulated in other sports [15, 24]. Finally, there was no consensus about the role of competition on the development of the decision‐making skills: some studies found association with the development of offensive decision‐making skills [14], while others found no association [15, 24, 37].

Overall, these findings suggest that high involvement in soccer‐specific activities is essential to develop decision‐making skills in soccer compared to non‐specific activities in other sports. Deliberate play has an important role, especially during childhood, to develop both decision‐making quality and speed [65], but also during early adolescence [15, 24, 65], while deliberate practice contributes to the development of decision‐making skills during childhood and adolescence [13, 14, 38, 65]. At the same time, these conclusions reflect patterns of association derived from players' developmental histories and should be interpreted as describing conditions under which decision‐making skills are more likely to emerge rather than prescriptive training solutions. It is important to note that, in most developmental studies, reported activity exposure reflects past engagement across childhood or adolescence, whereas decision‐making outcomes are typically assessed at later developmental stages. Altogether, these results provide support for participation in high amounts of soccer‐specific activities like deliberate play and practice since childhood to develop decision‐making skills. Therefore, it partially supports the early engagement pathway proposed by Ford et al. [72] and the specialized sampling pathway proposed by Sieghartsleitner et al. [73].

### 4.2 Interventions

#### 4.2.1 Studies With School Players

Most studies conducted with school players assessed the effectiveness of a hybrid intervention based on the Sport Education plus Invasion Games Competence model (*n* = 3) or Sports Education (*n* = 1), with sessions varying between 16 and 22. All of them were carried out with children and found a positive impact of such intervention on perceptual‐motor decision‐making skills for offensive situations [39, 40, 41], defensive situations [40, 41], and overall performance [39, 42]. In particular, both Farias et al. [39, 40] reported effect size measures, with moderate‐to‐large magnitudes for key decision‐making components and overall game performance (*r* ≈ 0.52–0.58; η^2^ ≈ 0.40–0.89), indicating that the observed improvements were not only statistically significant but also practically meaningful in school‐based interventions. Moreover, such studies showed that skill level [41] and sex [40, 41] were moderate factors of intervention effectiveness, indicating that the stimulus must be individualized to the different needs of children within the same group or classrooms [74].

Another four studies in this review investigated the effectiveness of intervention focusing on more tactical aspects of the game through the tactical games approach (*n* = 2), games approach (*n* = 1), and situated game teaching through set plays (*n* = 1) compared to technical approaches. No consensus about the effectiveness of interventions was found, as three of them [43, 45, 46] showed improvements in perceptual‐motor decision‐making skills in situations with the ball and one off‐the‐ball for the tactical intervention [43], while one showed no improvements [44]. Importantly, Gouveia et al. [45] reported small‐to‐moderate effect sizes for decision‐making, indicating practically meaningful effects of the tactical games approach. In contrast, although Garcia‐Ceberino et al. [44] reported effect size estimates, these were not accompanied by significant between‐group differences, and other studies did not report effect sizes [46]. This inconsistent result is probably due to the difference in the number of intervention sessions. The study from Garcia‐Ceberino et al. [44] applied only nine sessions, while the other three applied 10, 15, and 24 sessions. From a practical standpoint, these findings suggest that intervention duration may be a relevant factor, although heterogeneity across studies limits more precise conclusions regarding dose–response relationships. It suggests that more than nine sessions are needed to develop decision‐making skills through a tactical approach intervention.

Two studies in this review investigated the active role of students during an intervention, such as verbal interaction during training (play–discuss–play) [47] and students as peer teachers [48]. Both studies showed positive effects on perceptual‐motor decision‐making skills for the verbal interaction group and the peer‐teacher group in overall (offensive and defensive situations) decision accuracy. Taken together, findings from these studies indicate that instructional approaches that actively engage students are associated with small‐to‐moderate, practically meaningful improvements in perceptual‐motor decision‐making. Such results reinforce the importance of players performing an active and central role in sports training programs [75].

Finally, one study assessed the effectiveness of alternating teaching units of soccer and basketball compared to a traditional teaching unit of soccer and basketball to improve perceptual‐motor decision‐making skills [49]. The study found improvements in overall decision‐making in invasion sports, but not specific to soccer during the 10 sessions. Effect size analyses indicated small‐to‐moderate magnitudes for decision‐making at the level of overall invasion sports, whereas soccer‐specific decision‐making outcomes did not show significant effects over the intervention period. This pattern suggests that alternated teaching units may support more general decision‐making skills transferable across invasion sports, while their impact on soccer‐specific decision‐making appears limited under short‐term exposure. It suggests that more specific training, focusing on soccer content, is needed to develop decision‐making skills in soccer [68]. Importantly, these findings should be interpreted within the broader context of school‐based physical education, where interventions are typically designed to pursue multiple educational objectives, such as engagement, learning, and game understanding, rather than exclusively targeting improvements in game performance through decision‐making.

#### 4.2.2 Studies With Academy Players

Most studies in this review investigated the effectiveness of TGfU intervention (*n* = 4) on decision‐making skills. Improvements in perceptual‐motor skills were reported in three out of four studies related to offensive actions without the ball [50] and in offensive actions with the ball in passing situations [27, 51] and dribbling situations [27]. Importantly, Sierra‐Ríos et al. [50] explicitly reported medium‐to‐large effects for several decision‐making outcomes, whereas effect size measures were not consistently reported across the remaining TGfU studies, which limits the interpretation of the overall magnitude and practical relevance of the observed improvements. The study by Harvey et al. [52] showed no improvements in decision‐making skills related to offensive and defensive actions with the ball and was conducted using an uncontrolled within‐group design, without a comparison group. As such, its findings should be interpreted with caution and are not directly comparable to those derived from experimental or quasi‐experimental TGfU interventions. This study also involved fewer training sessions (*n* = 8), whereas other TGfU interventions ranged from 12 to 22 sessions. Moreover, it was the only study carried out with adolescents that assessed decision‐making skills through the GPAI instrument, which assesses more general aspects of game performance. On the other hand, the other three studies were carried out with children and used the GPET instrument, which assesses more specific principles (operational) of soccer than GPAI [76]. Overall, TGfU interventions implemented over more than eight sessions were more frequently associated with improvements in decision‐making skills when assessed with soccer‐specific instruments, although inconsistent effect size reporting limits conclusions regarding dose–response relationships.

These findings are consistent with evidence suggesting that learning environments characterized by representative task design, appropriate constraint manipulation, and continuous player engagement are more likely to promote transferable decision‐making skills. In this regard, O'Connor et al. [77] highlight that practice designs preserving the informational properties of the competitive environment provide more favorable conditions for the development of perceptual‐cognitive and perceptual‐motor decision‐making in team sports.

In terms of non‐linear pedagogy interventions, three studies found a positive effect on players' perceptual‐motor skills. The two studies that assessed decision‐making through competitive matches found improvements in passing situations [26, 54], but not in dribbling situations [54]. Moreover, Roberts et al. [53] showed improvements in decision‐making in shooting situations assessed throughout a shooting skill task. Notably, Práxedes et al. [54] reported a small overall effect size at the multivariate level, whereas Práxedes et al. [26] and Roberts et al. [53] did not report standardized effect size measures for decision‐making outcomes. These results also indicate that non‐linear pedagogy effectively develops different perceptual‐motor skills related to actions with the ball, like passing and shooting, but not for dribbling situations. Across the intervention durations examined, non‐linear pedagogy programs implemented over approximately 14 sessions were more frequently associated with improvements in passing‐ and shooting‐related decision‐making than in dribbling situations. However, given that effect size reporting was either limited to a small overall effect [54] or absent in the remaining studies, definitive conclusions regarding whether longer or more intensive interventions would lead to meaningful changes in dribbling‐related decision‐making remain premature.

Regarding interventions employing SSG based on core tactical principles and player interaction, two studies found a positive effect on players' perceptual‐cognitive decision‐making skills. Barcellos et al. [55] demonstrated improved decision‐making skills related to offensive tactical principles far from the ball, assessed through video analysis using the TacticUP instrument after 25 sessions. Moreover, another study [25] found improvements in perceptual‐cognitive decision‐making time for both offensive and defensive actions performed near the ball, and defensive actions performed far from the ball, assessed through the TacticUP instrument. They also found improvement in perceptual‐motor decision‐making skills for defensive actions performed near the ball. Machado, González‐Víllora, Roca, and Teoldo [25] reported medium‐to‐large effects for decision‐making outcomes, whereas Barcellos et al. [55] did not report effect sizes. These results suggest that SSG with a focus on individual development of players can also enhance the speed and efficiency of decision‐making in more congested and dynamic areas of the field, potentially due to the increased cognitive demands and need for rapid responses [5, 78]. However, variability in effect size reporting across studies warrants caution when interpreting the magnitude of SSG‐related improvements.

Five additional intervention types have been identified, comprising three field‐based and two laboratory‐based interventions. In terms of field‐based interventions, the study conducted by Valencia‐Sánchez and Arias [59] showed that the didactic model game action competencies (DMGAC) improved players' decision‐making skills considering the overall performance in offensive with and without the ball and defensive situations, assessing the core tactical principles of soccer in children compared to a direct instruction didactic model. Moreover, Práxedes et al. [60] found that an intervention with small‐sided games (SSG) with numerical superiority could develop offensive decision‐making skills related to passing situations of average‐level players, whereas an SSG intervention with numerical equality did not lead to improvements in either average‐ or low‐level players. Similarly, Villaseca‐Vicuña et al. [58] reported improvements in technical‐decisional performance following an integrated warm‐up protocol based on SSG, with a small effect size explicitly reported for decision‐making outcomes. Taken together, these field‐based findings indicate the importance of task design and player skill level when targeting decision‐making development, although the magnitude of effects is generally small or inconsistently reported across studies, reinforcing the need for careful individualization of training tasks [79].

Regarding laboratory‐based interventions, Feria‐Madueño et al. [57] demonstrated that a 6‐week attention training program, delivered via a video game prior to football training sessions, significantly enhanced players' perceptual‐motor decision‐making skills. These improvements were observed across various game situations, encompassing both offensive (with and without ball possession) and defensive scenarios, as measured by the GPET instrument. These findings suggest that optimizing attentional readiness prior to training may be an important factor in augmenting decision‐making skills. However, a similar study by Romeas et al. [56], which employed a 10‐week 3D MOT training program, found no significant improvements in players' perceptual‐motor offensive decision‐making skills as assessed by the GPAI instrument. These contrasting findings highlight the limited and inconsistent transfer of laboratory‐based perceptual‐cognitive training to on‐field decision‐making performance. While both studies share similarities in the nature of their interventions, the use of different assessment instruments, with GPET representing a football‐specific tool and GPAI assessing more general principles applicable to invasion games, may have played a central role in the divergent findings. This, in turn, warrants caution when interpreting the practical applicability of laboratory‐based decision‐making training [76].

Overall, these studies suggest that different Game‐based Approaches such as TGfU, non‐linear pedagogy, DMGAC, and SSG can contribute to the development of perceptual‐motor and perceptual‐cognitive decision‐making skills in academy players, particularly for offensive actions with the ball. However, the strength of this evidence is not uniform across approaches or study designs. Medium‐to‐large effects were reported in some field‐based interventions using soccer‐specific assessments; small effects were reported in others, and several studies did not report standardized effect sizes. In contrast, laboratory‐based interventions generally showed more inconsistent transfer to on‐field decision‐making and did not report standardized effect sizes, further limiting inferences about their practical relevance. This heterogeneity highlights the importance of interpreting intervention effects in light of both their reported magnitude and their methodological context, rather than assuming equivalent effectiveness across approaches.

Taken together, the pattern emerging across these different field‐based methodologies suggests that, in addition to the specific features of each pedagogical approach, their shared contribution to decision‐making development may be explained by common principles of practice design, such as task representativeness, constraint manipulation, and sustained player engagement in game‐like contexts, as highlighted in contemporary frameworks on learning environments and player development [71, 77, 80]. This interpretation is consistent with the learning environment perspective proposed by O'Connor et al. [77], who argue that environments preserving the informational and temporal demands of performance are more likely to support transferable decision‐making skills. Accordingly, the convergent findings observed across TGfU, non‐linear pedagogy, and small‐sided game interventions can be understood as reflecting common underlying learning principles rather than isolated methodological effects.

Moreover, assessing the effectiveness of interventions is necessary to use instruments adequate to players' age and sports development phases [76]. For instance, the literature suggests that during childhood (6–12 years), instruments that assess more general aspects of the soccer game, such as KORA [81] (general tactical principles) and GPET [82] (operational tactical principles) should be used [79]. Additionally, from the end of childhood (around 10 years onward) to older ages (adolescence and adulthood), more specific aspects of soccer should be assessed, like the core tactical principles and specific tactical principles. Instruments like TacticUP [74] and FUT‐SAT [83] allow the assessment of perceptual‐cognitive and perceptual‐motor skills, respectively, related to the core tactical principles, while game performance analysis could be used to assess specific tactical principles [79]. From this perspective, decision‐making development can be understood not merely as improvements in isolated responses, but as the progressive acquisition of understanding, reflected in players' ability to perceive affordances and flexibly apply tactical principles across contexts, as highlighted by Price and Collins [84]. Consequently, the use of age‐ and context‐appropriate instruments is essential to capture not only decision accuracy, but also transfer, adaptability, and contextualized decision‐making.

#### 4.2.3 Studies With University and Adult Players

The three studies conducted on university players found no consensus about the effectiveness of interventions. Two of them assessed the effectiveness of a laboratory 3D Multiple Object Tracking (3D MOT) training. Romeas et al. [62] found improvements in perceptual‐motor decision‐making accuracy in passing situations, with a medium effect size reported for this outcome, but not in dribbling and shooting, for which small or non‐significant effects were observed. On the other hand, Harenberg et al. [61] found no improvements in perceptual‐cognitive decision‐making skills in passing situations after conducting the same training program as Romeas et al. [62]. These contradictory results probably relate to the different methods used to assess decision‐making skills. While Romeas et al. [62] assessed perceptual‐motor skills with SSG, Harenberg et al. [61] assessed perceptual‐cognitive skills through a video‐based test. The use of different assessment instruments, combined with the limited ecological validity of laboratory‐based protocols, may partly explain the divergent findings and constrain direct translation to applied soccer contexts [74].

Another study, from Psotta and Martin [63], assessed the effectiveness of a combined tactical model training on perceptual‐motor skills related to passing, dribbling, and shooting decision‐making skills. It was found that improvements were made for passing situations but not for dribbling and shooting situations, similar to other studies carried out with university players [62] and soccer academy players [51, 54, 60]. However, Psotta and Martin [63] did not report standardized effect size measures, which limits conclusions regarding the magnitude and practical relevance of these improvements. Therefore, decision‐making skills related to passing situations apparently evolve quickly compared to dribbling and shooting skills, although the absence of effect size reporting prevents definitive conclusions about the relative magnitude of these effects.

Among adult professional athletes, the potential for cognitive training to enhance decision‐making skills is worthy of further investigation. Fortes et al. [64] provide evidence supporting this notion, finding that transcranial direct current stimulation (tDCS) improved perceptual‐cognitive decision‐making speed in offensive situations with the ball, with moderate effect sizes reported for response–time outcomes, but not perceptual‐cognitive or perceptual‐motor decision‐making accuracy in soccer players. While these findings suggest that interventions targeting cognitive processes may offer a practical approach to enhancing reaction time, they provide limited evidence for meaningful improvements in applied decision‐making accuracy, and substantial caution is warranted given the reliance on a single study, the laboratory‐based nature of the intervention, and the restricted scope of observed effects.

### 4.3 Limitations

Some limitations of the present review should be acknowledged. First, the body of evidence addressing developmental activities was relatively limited, with only seven studies identified, and studies including female participants were restricted to a single country. This limits the generalizability of the findings and highlights an important gap in the literature regarding sex‐specific and cross‐cultural perspectives on decision‐making development in soccer.

In addition, most studies focused primarily on the quality of decision‐making, whereas only one study examined decision‐making speed. This represents a relevant limitation, given that decision‐making quality and speed are conceptually distinct and complementary components of performance [67], particularly in time‐pressured environments such as competitive soccer. The predominance of quality‐based measures may therefore provide an incomplete picture of decision‐making development.

The geographical distribution of the included studies was also uneven, with most research conducted in a small number of countries. Considering that developmental pathways and training environments are influenced by cultural, organizational, and contextual factors, this concentration may further limit the transferability of the findings to other soccer contexts [85]. Finally, several studies relied on subjective assessments of decision‐making, underscoring the need for a broader use of objective, standardized, and soccer‐specific measurement approaches in future research.

## 5 Conclusions

This study was the first to scope peer‐reviewed literature on: (i) developmental activities and their contribution to the development of decision‐making skills in soccer players and (ii) the effect of interventions on soccer players' decision‐making skills. Our findings suggest that high involvement in soccer‐specific activities is essential to developing decision‐making skills compared to non‐specific activities in other sports. Furthermore, deliberate play has an important role, especially during childhood, but also during early adolescence [15, 24, 65], while deliberate practice contributes to the development of decision‐making skills during childhood and adolescence [13, 14]. Moreover, practice in futsal during childhood and early adolescence has been linked to enhanced decision‐making skills [38, 65]. However, subsequent research suggests that the key is not simply accumulating hours of deliberate play or deliberate practice [38, 65]. Rather, a specific relationship between deliberate play and practice during childhood, characterized by a balanced combination, followed by a strategic shift towards increased structured practice in early adolescence, may be crucial for developing elite players, much like the carefully balanced ingredients in a successful recipe, where the right proportions are essential for optimal results [36].

In terms of interventions, we found that Game‐based Approaches such as TGfU, non‐linear pedagogy, small‐sided games, Invasion Games Competence model, situated game teaching through set plays, and Tactical Games Approach can effectively develop some aspects of perceptual‐motor decision‐making skills in different contexts, like school players, soccer academy players, and university players. Moreover, the effectiveness of laboratory interventions to develop decision‐making skills in soccer players is not guaranteed, as little research was conducted on this topic [56, 57, 61, 62, 64], and they found contradictory results. Finally, the limitations of the current literature were discussed, and future directions were presented.

## 6 Perspective

Future research should seek to broaden the scope of evidence on decision‐making development by including more diverse samples, particularly female players and adult populations, which remain underrepresented in both developmental and intervention‐based studies [85]. Expanding research across different cultural and competitive contexts may also help clarify how contextual factors shape decision‐making acquisition and performance in soccer [85].

Greater attention should be given to decision‐making speed, especially in intervention studies, as this variable has received limited empirical focus despite its practical relevance in match play [67]. Longitudinal and experimental designs that jointly examine decision‐making quality and speed may provide a more comprehensive understanding of how these components evolve across developmental stages [67].

Regarding interventions, future studies would benefit from systematically examining the role of intervention duration and structure, as most positive outcomes were observed in programs lasting 12 sessions or more [56]. In addition, further investigation is needed to clarify how different practice designs and learning environments influence decision‐making development across age groups and levels of expertise [72, 73]. Finally, examining related invasion sports and alternative developmental pathways may offer additional insights into the mechanisms underpinning decision‐making skill acquisition in soccer and contribute to refining existing developmental models [72, 73].

## Funding

This study received support from CNPq, FAPEMIG, CAPES, Funarbe, SEESP‐MG, the Dean’s Office for Graduate and Research Studies, and the Centre of Life and Health Sciences at the Federal University of Viçosa, Brazil. Additionally, funding for this research was provided in part by the National Secretariat of Football and Fan Rights (SNFDT) through the Academy & Football Program, and by grants from CNPQ, Conselho Nacional de Desenvolvimento Científico e Tecnológico and Coordenação de Aperfeiçoamento de Pessoal de Nível Superior‐ Brasil (CAPES) – Finance Code 001.

## Conflicts of Interest

The authors declare no conflicts of interest.

## Data Availability

Data sharing not applicable to this article as no datasets were generated or analysed during the current study.
